# Reactogenicity after heterologous and homologous COVID-19 prime-boost vaccination regimens: descriptive interim results of a comparative observational cohort study

**DOI:** 10.1186/s12879-022-07443-x

**Published:** 2022-05-31

**Authors:** Lisette Warkentin, Nikoletta Zeschick, Thomas Kühlein, Philipp Steininger, Klaus Überla, Isabelle Kaiser, Christine Gall, Maria Sebastião, Susann Hueber

**Affiliations:** 1 Institute of General Practice, Friedrich-Alexander-Universität Erlangen-Nürnberg, University Hospital Erlangen, Universitätsstraße 29, Erlangen, Germany; 2 Institute of Clinical and Molecular Virology, Friedrich-Alexander-Universität Erlangen-Nürnberg, University Hospital Erlangen, Schloßgarten 4, Erlangen, Germany; 3 Department of Medical Informatics, Biometry and Epidemiology, Friedrich-Alexander-Universität Erlangen-Nürnberg, Waldstraße 6, Erlangen, Germany

**Keywords:** COVID-19, Vaccines, Adverse drug reaction, Observational study, Survey

## Abstract

**Background:**

Due to safety signals after vaccination with COVID-19 vector vaccines, several states recommended to complete the primary immunization series in individuals having received one dose of ChAdOx1 (AstraZeneca) with an mRNA vaccine. However, data on safety and reactogenicity of this heterologous regimen are still scarce. The aim of this study was therefore to compare the reactogenicity and the frequency of medical consultations after boost vaccination in a heterologous regimen with ChAdOx1 and mRNA-vaccines (BNT162b2, BioNTech/Pfizer or mRNA-1273, Moderna) to homologous regimens with ChAdOx1 or mRNA-vaccines, respectively.

**Methods:**

In an observational cohort study reactogenicity and safety were assessed 14–19 days (short-term) and 40 to 56 days (long-term) after the boost vaccination using web-based surveys. In the short-term survey solicited and unsolicited reactions were assessed, while the long-term survey focussed on health problems leading to medical consultation after the vaccination, including those that were not suspected to be vaccine-related.

**Results:**

In total, 9146 participants completed at least one of the surveys (ChAdOx1/ChAdOx1: *n* = 552, ChAdOx1/mRNA: *n* = 2382, mRNA/mRNA: *n* = 6212). In the short-term survey, 86% with ChAdOx1/mRNA regimen reported at least one reaction, in the ChAdOx1/ChAdOx1 and mRNA/mRNA cohorts 58% and 76%, respectively (age and sex adjusted *p* < 0.0001). In the long-term survey, comparable proportions of individuals reported medical consultation (ChAdOx1/ChAdOx1 vs. ChAdOx1/mRNA vs. mRNA/mRNA: 15% vs. 18% vs. 16%, age and sex adjusted *p* = 0.398). Female gender was associated with a higher reactogenicity and more medical consultations. Younger age was associated with a higher reactogenicity, whereas elderly people reported more medical consultations.

**Conclusion:**

Although the short-term reactogenicity was higher with the heterologous regimen than with the homologous regimens, other factors such as higher efficacy and limited resources during the pandemic may prevail in recommending specific regimens.

## Background

The efficacy and safety of the vaccines BNT162b2 (BioNTech/Pfizer), mRNA-1273 (Moderna), and ChAdOx1 (AstraZeneca) have been demonstrated in large randomized controlled trials [1–3]. Administration of ChAdOx1 started in February 2021 in EU/EEA countries [4]. As of March 2021, an association between ChAdOx1 administration and the occurrence of thromboembolic events, later referred to as vaccine-induced immune thrombotic thrombocytopenia (VITT), has been detected [4–6]. This safety signal led to different recommendations in EU/EEA countries: while some restricted the administration of ChAdOx1 to the elderly as done in Germany, others suspended its use completely [4]. As many people were already primed with ChAdOx1, some countries recommended a boost with BNT162b2 or mRNA-1273 [7–10]. In Germany, this recommendation was first restricted to persons younger than 60 years. By July 2021 this heterologous regimen was extended to all individuals due to safety concerns and because initial data indicated an even better immunogenicity [8, 11]. Additionally, preclinical trials investigating the immune responses after different heterologous vaccination regimens show promising results [12–15]. However, evidence on the safety and reactogenicity of the heterologous vaccination regimen was still scarce. Several studies have been published, though mostly with small sample sizes, a maximal follow-up time of two weeks, and, in some studies, a control group with a homologous regimen was missing [16–22]. The reactogenicity of the heterologous vaccination regimen reported in these studies was comparable to or higher than that of the homologous vaccination regimens.

The safety study reported here is embedded in the CoVaKo project (*Corona Vakzin Konsortium*) that analyses the efficacy and safety of COVID-19 vaccines [23, 24]. In the CoVaKo safety study we aim to monitor reactogenicity and health problems after COVID-19 vaccination compared to other vaccinations like influenza or pneumococcal vaccination. A longitudinal online survey was used with the focus on health problems occurring within 18 weeks after vaccination and leading to medical consultation, medication intake, or sick-leave. Due to potential shortages of particular vaccines, practical considerations in the absence of single dose vaccine vials, and the potential need for a booster after initial immunization, in particular after vaccination with COVID-19 vector vaccines, evaluating the safety of heterologous vaccination regimens became very important as well. Therefore, this interim analysis of the safety study focusses on the comparison of reactogenicity and health problems after the second dose in a heterologous regimen with ChAdOx1 as prime and an mRNA-vaccine as boost in comparison to homologous regimens with ChAdOx1 or mRNA-vaccines, respectively.

## Methods

### Study design and setting

In an observational cohort study, reactogenicity and safety of vaccinations were assessed including 14 to 19 days (short-term survey) and 40 to 59 days (long-term survey) after the second COVID-19 vaccination using web-based surveys [25]. In an interim analysis reactions and health problems after the second dose were compared in individuals with (1) homologous immunization with ChAdOx1 (ChAdOx1/ChAdOx1), (2) heterologous immunization with ChAdOx1 as first dose and BNT162b2 or mRNA-1273 as second dose (ChAdOx1/mRNA), or (3) homologous immunization with mRNA vaccination BNT162b2 or mRNA-1273 (mRNA/mRNA).

Recruitment of vaccinated participants commenced on April 17, 2021 in vaccination centres and primary care practices in Bavaria, Germany. The data collection period for the interim analysis ended on August 16, 2021. After a vaccination, individuals received a leaflet with information on the study and had the possibility to voluntarily register on a web-based platform. Afterwards they received the links to the short- and long-term surveys. The recruitment strategy and the surveys were evaluated in a feasibility study (registered at DRKS: ID DRKS00025881). Recruitment for the main study started on May 20, 2021 and is planned to be continued until January 2022 (registered at DRKS: ID DRKS00025373) [23]. Due to the dynamic changes in vaccination regimens, and the importance of generating real-world evidence on the safety of the different prime-boost regimens, we included both, the data of the feasibility study and the main study in this interim analysis. With only minor changes to the survey between the feasibility study [25] and main study this approach was considered methodologically valid. All methods were carried out in accordance with relevant guidelines and regulations. The reporting of the study is based on the STROBE (Strengthening the Reporting of Observational studies in Epidemiology) recommendations [26].

### Participants and variables

Individuals born before the year of 2004 who received a vaccination (against COVID-19, influenza, pneumococcus, tickborne encephalitis, tetanus/diphtheria vaccination with or without pertussis/poliomyelitis, and/or herpes zoster) in the last 124 days were able to register for the safety study. After giving their informed consent on the web-based registration form, participants were asked about sociodemographic characteristics, comorbidities, and information on the vaccination including the brand name and batch number. Questions on morbidity were based on a modified German version of the Self-Administered Comorbidity Questionnaire (mSCQ-D) [27, 28]. In the short-term survey solicited and unsolicited local and systemic reactions were assessed. Solicited reactions were known reactions after vaccinations like local pain, headache, and fever. Participants were able to report unsolicited reactions in a free text field. The reactions were determined along with possible consequences like medical consultation, medication intake, or sick-leave. The long-term survey focussed only on health problems leading to in- or outpatient medical consultation. In order to classify health problems in relation to the vaccination, participants were asked to indicate all events that occurred in the respective time interval and then to rate if they suspected an association to the vaccination (Additional file, survey). Data is collected using the web-based software platform REDCap (Research Electronic Data Capture), hosted at Universitätsklinikum Erlangen [29, 30].

### Statistical analysis

**Fig. 1:**
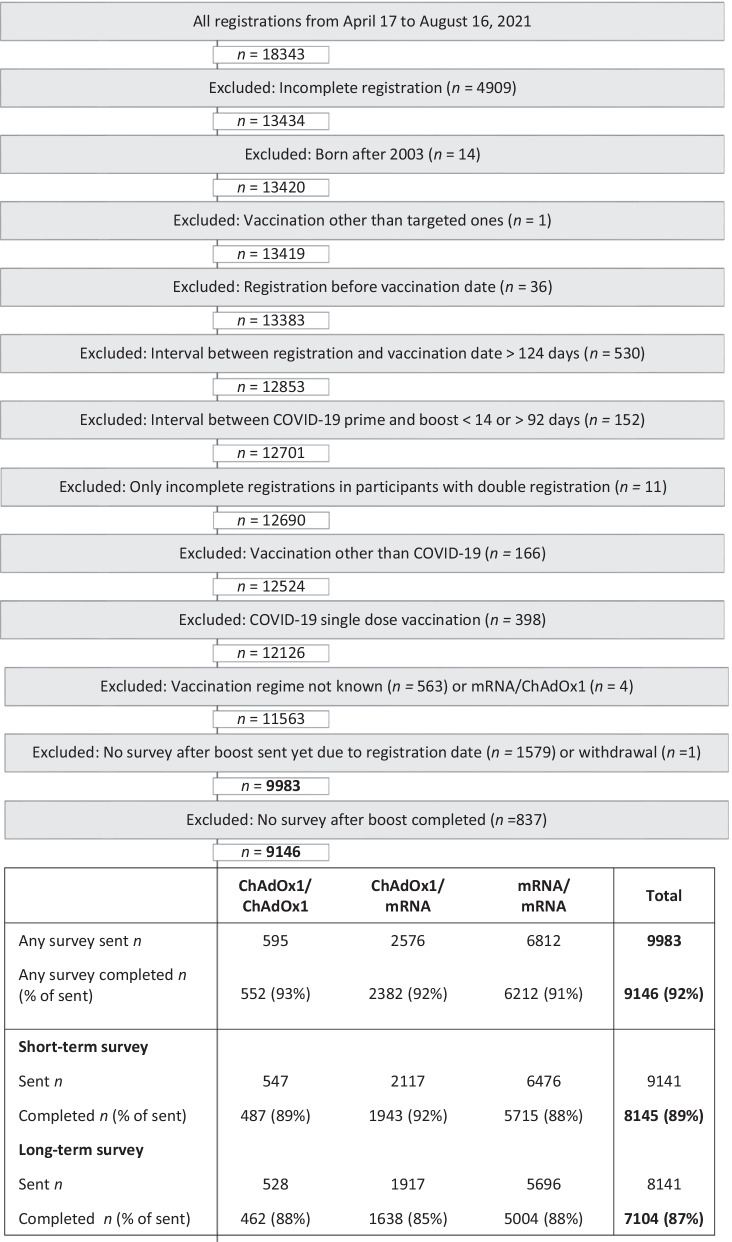
Data selection process and response rates

Participants who received an email with a personalized link to the short- and/or long-term survey during the data collection period were selected for the interim analysis. Participants with incomplete registrations were excluded. Further exclusion criteria were birth years after 2003, having received none of the targeted vaccinations, registration before vaccination date or later than 124 days after vaccination of first or single dose, and an interval between prime and boost of less than 14 days or more than 92 days. Email addresses were checked for duplicates. In case one person registered twice, the datasets were synthesized. If one email address was used by two persons, both datasets were considered separately. For plausibility we checked, whether the invitation links were sent at the correct time in regard to the vaccination date. If sent at an incorrect time, answers were set to missing. In case of implausible age (year of birth before 1900), weight (lower than 20 kg or higher than 300 kg), height (lower than 50 cm or higher than 250 cm), and/or pregnancy (in male participants or participants with birth year before 1975), the respective variables were set to missing. For the interim analysis, only participants having received prime and boost COVID-19 vaccination with known vaccination regimen were selected. Data of participants who completed at least one of the surveys is analysed. After the selection process, 9146 participants remained (Fig. 1).

The response-rate is reported as proportion of fully completed and valid surveys to all surveys sent out to the participants that remained after data selection process. As age was reported as year of birth, it was calculated as the difference between the year 2021 and the year of birth provided. Sociodemographic characteristics, comorbidities, and interval between prime and boost vaccination are reported as proportion or as a mean/median. Comorbidity in form of mSCQ-D was calculated. Consequences of reactions were queried in a multiple-choice question. In the descriptive analysis they were ordered hierarchical. The consequence perceived as most serious is reported (from no consequence to medication intake, sick leave, outpatient (practice) consultation, clinic (ambulant) consultation, and hospitalisation). Health problems are reported as absolute and relative frequencies. Due to the COVID-19 vaccine prioritization the participants were not distributed equally throughout the cohorts especially regarding age and gender. Therefore, results are also reported separately for gender and age with a threshold of 55 years chosen according to other studies on COVID-19 vaccines [1, 2, 16]. Furthermore, group comparisons with respect to the rate of overall reactions/health problems in the short-term and long-term survey were analysed by logistic regression adjusted by age (continuous, non-dichotomized) and gender with overall *p*-values from ANOVA.

Data preparation, analyses and figures were performed using R Statistical Software (version 4.0.2, R Foundation for Statistical Computing, Vienna, Austria).

## Results

### Sociodemographic characteristics

In total, at least one survey was sent to 9983 participants, out of whom 92% responded to at least one survey and completed it validly (*n* = 9146). The short-term survey was completed validly by 8145 (89%) and the long-term survey by 7104 (87%). The cohorts for the short- and long-term survey are described separately. The ChAdOx1/ChAdOx1 cohorts were the smallest, followed by ChAdOx1/mRNA and mRNA/mRNA (Fig. 1). The ChAdOx1/ChAdOx1 cohorts had a higher mean age, a higher proportion of male participants and a higher proportion of people with at least one comorbidity as compared to the other cohorts (Table 1). In contrast, no major difference was detected in the mean age and proportion of participants with at least one comorbidity between the mRNA/mRNA and the ChAdOx1/mRNA cohorts. In the ChAdOx1/mRNA cohorts, the proportion of female participants was higher among respondents to the long-term survey than in respondents to the short-term survey (Table 1).

**Table 1:** Baseline characteristic of participants with a COVID-19 boost vaccination in a homologous or heterologous regimen

	Completed short-term survey			Completed long-term survey		
Baseline characteristics	ChAdOx1/ChAdOx1	ChAdOx1/mRNA	mRNA/mRNA	ChAdOx1/ChAdOx1	ChAdOx1/mRNA	mRNA/mRNA
*n*	487	1943	5715	462	1638	5004
**Interval prime-boost (median (IQR) in days)**	84 (63–84)	78 (63–84)	42 (42–42)	84 (63–84)	65 (63–84)	42 (42–42)
**Age (mean ± SD)**	55.87 ± 15.3	47.6 ± 13.89	45.87 ± 15.14	56.69 ± 15.61	45.1 ± 12.52	47.37 ± 15.23
**Gender**						
Male	272 (55.9%)	778 (40.0%)	2428 (42.5%)	257 (55.6%)	448 (27.4%)	2133 (42.6%)
Female	215 (44.1%)	1165 (60.0%)	3281 (57.4%)	205 (44.4%)	1189 (72.6%)	2865 (57.3%)
Diverse	0 (0%)	0 (0%)	6 (0.1%)	0 (0%)	1 (0.1%)	6 (0.1%)
**Residence**						
Rural area^1^	181 (37.2%)	597 (30.7%)	1867 (32.7%)	166 (35.9%)	485 (29.6%)	1676 (33.5%)
Small town^2^	158 (32.4%)	540 (27.8%)	1700 (29.7%)	147 (31.8%)	414 (25.3%)	1540 (30.8%)
Medium-sized town^3^	60 (12.3%)	236 (12.1%)	694 (12.1%)	59 (12.8%)	193 (11.8%)	607 (12.1%)
City^4^	88 (18.1%)	570 (29.3%)	1454 (25.4%)	90 (19.5%)	546 (33.3%)	1181 (23.6%)
**Employment**						
Employed	293 (60.2%)	1458 (75.0%)	3986 (69.7%)	265 (57.4%)	1347 (82.2%)	3426 (68.5%)
In education	13 (2.7%)	137 (7.1%)	576 (10.1%)	14 (3.0%)	115 (7.0%)	452 (9.0%)
Unemployed	9 (1.8%)	37 (1.9%)	147 (2.6%)	8 (1.7%)	22 (1.3%)	134 (2.7%)
Retired	163 (33.5%)	263 (13.5%)	803 (14.1%)	166 (35.9%)	105 (6.4%)	819 (16.4%)
Other	6 (1.2%)	36 (1.9%)	141 (2.5%)	5 (1.1%)	41 (2.5%)	120 (2.4%)
Not specified	3 (0.6%)	12 (0.6%)	62 (1.1%)	4 (0.9%)	8 (0.5%)	53 (1.1%)
**Education**						
No degree	0 (0%)	0 (0%)	8 (0.1%)	0 (0%)	0 (0%)	8 (0.2%)
Lower certificate	24 (4.9%)	52 (2.7%)	212 (3.7%)	23 (5.0%)	39 (2.4%)	202 (4.0%)
Intermediate certificate	88 (18.1%)	217 (11.2%)	695 (12.2%)	82 (17.7%)	184 (11.2%)	658 (13.1%)
Complete apprenticeship	108 (22.2%)	280 (14.4%)	951 (16.6%)	91 (19.7%)	241 (14.7%)	862 (17.2%)
High school diploma	71 (14.6%)	373 (19.2%)	1066 (18.7%)	66 (14.3%)	347 (21.2%)	918 (18.3%)
University degree	186 (38.2%)	994 (51.2%)	2665 (46.6%)	189 (40.9%)	800 (48.8%)	2249 (44.9%)
Not specified	10 (2.1%)	27 (1.4%)	118 (2.1%)	11 (2.4%)	27 (1.6%)	107 (2.1%)
**Health status**						
No pre-existing diseases	148 (30.4%)	764 (39.3%)	2161 (37.8%)	147 (31.8%)	641 (39.1%)	1792 (35.8%)
Allergies	119 (24.4%)	556 (28.6%)	1629 (28.5%)	115 (24.9%)	520 (31.7%)	1453 (29%)
Hypertension	164 (33.7%)	350 (18%)	1008 (17.6%)	140 (30.3%)	250 (15.3%)	976 (19.5%)
Backpain	83 (17.0%)	269 (13.8%)	831 (14.5%)	84 (18.2%)	250 (15.3%)	754 (15.1%)
Lung Disease	40 (8.2%)	110 (5.7%)	411 (7.2%)	37 (8.0%)	113 (6.9%)	387 (7.7%)
Rheumatism/Autoimmune disease	29 (6.0%)	125 (6.4%)	467 (8.2%)	31 (6.7%)	126 (7.7%)	428 (8.6%)
Depression	24 (4.9%)	90 (4.6%)	413 (7.2%)	25 (5.4%)	86 (5.3%)	359 (7.2%)
Osteoarthritis	43 (8.8%)	125 (6.4%)	324 (5.7%)	42 (9.1%)	97 (5.9%)	311 (6.2%)
Gastrointestinal disease	26 (5.3%)	104 (5.4%)	375 (6.6%)	26 (5.6%)	89 (5.4%)	341 (6.8%)
Heart disease	49 (10.1%)	61 (3.1%)	261 (4.6%)	45 (9.7%)	47 (2.9%)	254 (5.1%)
Diabetes	37 (7.6%)	70 (3.6%)	208 (3.6%)	32 (6.9%)	60 (3.7%)	204 (4.1%)
Cancer	25 (5.1%)	38 (2.0%)	127 (2.2%)	27 (5.8%)	43 (2.6%)	130 (2.6%)
Coagulation problems	11 (2.3%)	32 (1.6%)	144 (2.5%)	10 (2.2%)	41 (2.5%)	138 (2.8%)
Kidney disease	12 (2.5%)	30 (1.5%)	81 (1.4%)	12 (2.6%)	26 (1.6%)	81 (1.6%)
Liver disease	6 (1.2%)	17 (0.9%)	73 (1.3%)	7 (1.5%)	17 (1.0%)	69 (1.4%)
Anaemia	7 (1.4%)	17 (0.9%)	44 (0.8%)	6 (1.3%)	21 (1.3%)	35 (0.7%)
**mSCQ-D (median (IQR))**^**5**^	1 (0–2)	0 (0–2)	0 (0–2)	1 (0–2)	0 (0–2)	1 (0–2)
**BMI (mean ± SD)**^**6**^	27.0 ± 5.5 (*NA*^7^ = 7)	25.8 ± 5.2 (*NA* = 22)	26.1 ± 5.5 (*NA* = 64)	26.8 ± 5.4 (*NA* = 7)	25.8 ± 5.7 (*NA* = 17)	26.2 ± 5.6 (*NA* = 63)
**Participants with other vaccination(s)**^**8**^	30 (6.2%)	135 (6.9%)	456 (8.0%)	23 (5.0%)	97 (5.9%)	389 (7.8%)

Cohorts of participants with homologous (mRNA/mRNA or ChAdOx1/ChAdOx1) or heterologous (ChAdOx1/mRNA) prime-boost COVID-19 vaccination regimen who completed the short- and/or long-term survey. ChAdOx1: ChAdOx1 (AstraZeneca). mRNA: BNT162b2 (BioNTech/Pfizer) or mRNA-1273 (Moderna). ^1^Rural area =  < 5.000. ^2^Small town = 5.000 to approx. 20.000. ^3^Medium-sized town = 20.000 to approx. 100.000. ^4^City = 100.000 or more inhabitants. ^5^mSCQ-D = modified German version of the Self-Administered Comorbidity Questionnaire. ^6^BMI = Body Mass Index. ^7^NA = not applicable. ^8^Participants with other vaccination(s) received at least one other vaccination in an interval between eight weeks before their first COVID-19 vaccination and the long-term survey

### Short-term survey

At least one solicited or unsolicited reaction was reported by 86% of participants in the ChAdOx1/mRNA cohort and by 58% and 76% of participants in the ChAdOx1/ChAdOx1 and mRNA/mRNA cohort, respectively (adjusted *p* < 0.0001). Logistic regression showed lower reactogenicity in ChAdOx1/ChAdOx1 (OR = 0.303, 95% CI [0.240, 0.383]) and mRNA/mRNA (OR = 0.467, 95% CI [0.403, 0.541]) as compared to ChAdOx1/mRNA cohort.

**Fig. 2:**
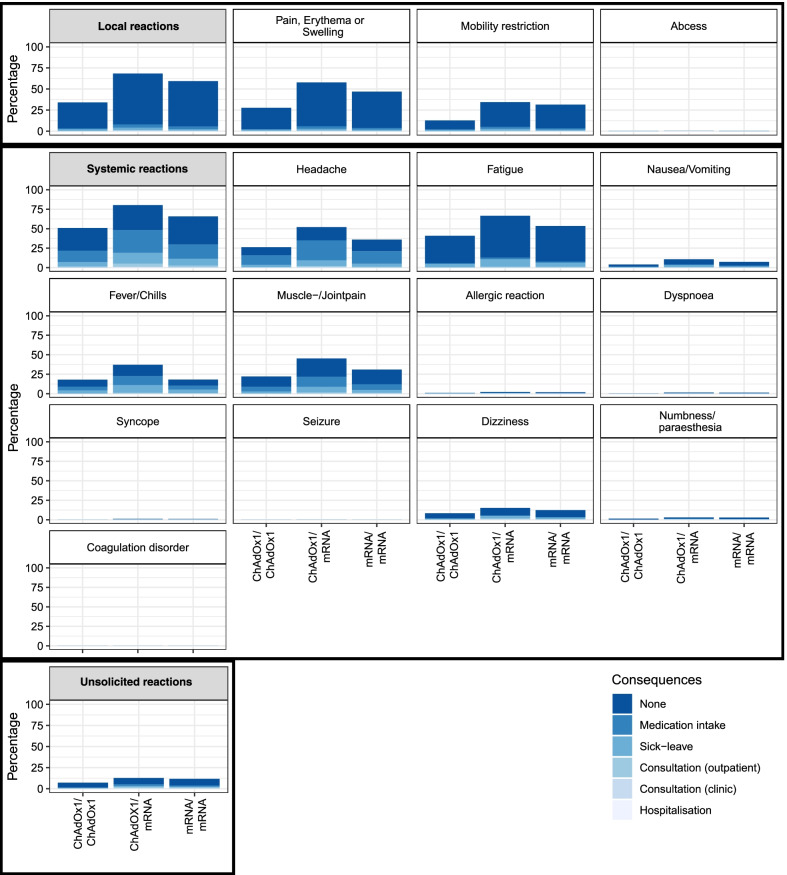
Reactions after boost COVID-19 vaccination in a homologous or heterologous regimen. Solicited and unsolicited reactions 14 to 19 days after boost COVID-19 vaccination in participants with homologous (mRNA/mRNA or ChAdOx1/ChAdOx1) or heterologous (ChAdOx1/mRNA) prime-boost vaccination regimen with consequences in hierarchical order, as multiple choice was possible. The consequence perceived as most serious is reported (from no consequence to medication intake, sick leave, outpatient (practice) consultation, clinic (ambulant) consultation, and hospitalisation). Local reactions are a composite of pain, erythema, or swelling, mobility restriction, and abscess. Systemic reactions are a composite of the reactions from headache to coagulation disorder. ChAdOx1: ChAdOx1 (AstraZeneca). mRNA: BNT162b2 (BioNTech/Pfizer) or mRNA-1273 (Moderna)

Participants with ChAdOx1/mRNA reported more local and systemic reactions than those with homologous regimens (local: ChAdOx1/ChAdOx1 34% vs. ChAdOx1/mRNA 68% vs. mRNA/mRNA 59%, systemic: ChAdOx1/ChAdOx1 51% vs. ChAdOx1/mRNA 80% vs. mRNA/mRNA 66%). Unsolicited reactions were reported almost equally after homologous and heterologous mRNA boost (ChAdOx1/ChAdOx1 7% vs. ChAdOx1/mRNA 13% vs. mRNA/mRNA 12%). Any consequence of reactions was most often reported in the ChAdOx1/mRNA group (ChAdOx1/ChAdOx1 41% vs. ChAdOx1/mRNA 58% vs. mRNA/mRNA 42%). Those consequences were mostly medication intake and sick leave (ChAdOx1/ChAdOx1 90% vs. ChAdOx1/mRNA 88% vs. mRNA/mRNA 87%). Out of all participants who experienced consequences, 42 participants reported a consultation in a clinic (ChAdOx1/ChAdOx1 0.9%, ChAdOx1/mRNA 1.8%, mRNA/mRNA 1.3%) and 18 participants reported an inpatient treatment (ChAdOx1/ChAdOx1 0.0%, ChAdOx1/mRNA 0.8%, mRNA/mRNA 0.6%). Results are depicted in Fig. 2.

No major difference was detected in the proportion of participants reporting at least one reaction when separating by interval between second dose of COVID-19 vaccination and registration to the study (Table 2). More participants in the ChAdOx1/mRNA group suspected an association between the vaccination and their reactions and felt affected by the reactions. In the mRNA/mRNA group 2.8% reported they suspect their reactions have long-term consequences, 2.4% in the ChAdOx1/mRNA group and 2.2% in the ChAdOx1/ChAdOx1 group, respectively. Participants with a homologous regimen perceived the vaccination more often as comparable to previous vaccinations (Table 2).

**Table 2:** Reactions and their perception 14 to 19 days after boost COVID-19 vaccination

	ChAdOx1/ChAdOx1	ChAdOx1/mRNA	mRNA/mRNA
	(*n* = 487)	(*n* = 1943)	(*n* = 5715)
Proportion of participants with at least one reported reaction			
Total	282 (57.9%)	1675 (86.2%)	4338 (75.9%)
< 1d interval vaccination – registration	131 (57.7%)	604 (84.2%)	2430 (74.9%)
1–5d interval vaccination – registration	112 (57.4%)	611 (86.1%)	1387 (76.3%)
> 5d interval vaccination – registration	39 (60.9%)	460 (89.1%)	521 (79.7%)

Perception	ChAdOx1/ChAdOx1	ChAdOx1/mRNA	mRNA/mRNA
	(*n* = 282, NA = 2)	(*n* = 1675)	(*n* = 4338, NA = 2)
**1) Suspected association between reactions and vaccination**			
Strongly disagree	0 (0.0%)	4 (0.2%)	23 (0.5%)
Disagree	7 (2.5%)	10 (0.6%)	89 (2.1%)
Agree	62 (22.1%)	194 (11.6%)	768 (17.7%)
Strongly agree	211 (75.4%)	1467 (87.6%)	3456 (79.7%)
**2) Affection by the reactions**			
Strongly disagree	29 (10.4%)	105 (6.3%)	412 (9.5%)
Disagree	75 (26.8%)	276 (16.5%)	1110 (25.6%)
Agree	99 (35.4%)	590 (35.2%)	1501 (34.6%)
Strongly agree	77 (27.5%)	704 (42.0%)	1313 (30.3%)
**3) Long-term consequences by the reactions**			
Strongly disagree	233 (83.2%)	1443 (86.1%)	3661 (84.4%)
Disagree	41 (14.6%)	193 (11.5%)	553 (12.8%)
Agree	3 (1.1%)	31 (1.9%)	79 (1.8%)
Strongly agree	3 (1.1%)	8 (0.5%)	43 (1.0%)

Participants with at least one reaction reported 14 to 19 days after boost COVID-19 vaccination (short-term survey) with ChAdOx1 or mRNA vaccine in cohorts of participants with homologous (mRNA/mRNA or ChAdOx1/ChAdOx1) or heterologous (ChAdOx1/mRNA) prime-boost COVID-19 vaccination regimen who completed the short-term survey. ChAdOx1: ChAdOx1 (AstraZeneca). mRNA: BNT162b2 (BioNTech/Pfizer) or mRNA-1273 (Moderna). *NA* not applicable

### Long-term survey

In the period since the short-term survey, 18% of the heterologous group reported any medical consultation as compared to 15% in the ChAdOx1/ChAdOx1 and 16% in the mRNA/mRNA group (adjusted *p* = 0.398). Of those, 97% to 99% reported (planned) outpatient consultation. Hospital admission was most frequently reported by the ChAdOx1/ChAdOx1 cohort and fewest by the heterologous group (ChAdOx1/ChAdOx1 19% vs. ChAdOx1/mRNA 7% vs. mRNA/mRNA 11%, Table 3). The health problems leading to medical consultations were in 13% to 16% pre-existing conditions and in 32% to 37% at least partially pre-existing conditions. In the ChAdOx1/ChAdOx1 cohort, 81% of individuals with medical consultation did not assume an association between the vaccination and their conditions, as compared to 74% in the other two cohorts.

**Table 3:** Medical consultation and health problems 40 to 56 days after boost COVID-19 vaccination

	ChAdOx1/ChAdOx1	ChAdOx1/mRNA	mRNA/mRNA
	(*n* = 462)	(*n* = 1638)	(*n* = 5004)
**Medical consultation, thereof…**	**69 (14.9%)**	**287 (17.5%)**	**796 (15.9%)**
**Outpatient**			
Yes	61 (88.4%)	248 (86.4%)	667 (83.8%)
Not yet, but planned	6 (8.7%)	36 (12.5%)	117 (14.7%)
No	2 (2.9%)	3 (1.0%)	12 (1.5%)
**Inpatient/Clinic**			
Yes	13 (18.8%)	21 (7.3%)	85 (10.7%)
Not yet, but planned	1 (1.4%)	2 (0.7%)	9 (1.1%)
No	55 (79.7%)	264 (92%)	702 (88.2%)
**Pre-existing conditions**			
Yes	11 (15.9%)	40 (13.9%)	103 (12.9%)
Partially	22 (31.9%)	95 (33.1%)	291 (36.6%)
No	36 (52.2%)	152 (53%)	402 (50.5%)
**Suspected association to vaccination**			
Yes	3 (4.3%)	16 (5.6%)	36 (4.5%)
Partially	10 (14.5%)	60 (20.9%)	171 (21.5%)
No	56 (81.2%)	211 (73.5%)	589 (74.0%)
**Perceived affection by the health problems**			
Strongly disagree	6 (8.7%)	24 (8.4%)	72 (9%)
Disagree	7 (10.1%)	38 (13.2%)	88 (11.1%)
Agree	21 (30.4%)	108 (37.6%)	326 (41%)
Strongly agree	35 (50.7%)	117 (40.8%)	310 (38.9%)
**Perceived long-term consequences by the health problems**			
Strongly disagree	20 (29.0%)	98 (34.1%)	237 (29.8%)
Disagree	28 (40.6%)	104 (36.2%)	256 (32.2%)
Agree	15 (21.7%)	54 (18.8%)	212 (26.6%)
Strongly agree	6 (8.7%)	31 (10.8%)	91 (11.4%)

	ChAdOx1/ChAdOx1	ChAdOx1/mRNA	mRNA/mRNA
	(*n* = 462)	(*n* = 1638)	(*n* = 5004)
Health problems leading to medical consultation, thereof…			
** Musculoskeletal disorders**	**35 (7.6%)**	**123 (7.5%)**	**321 (6.4%)**
Muscle weakness	5 (1.1%)	14 (0.9%)	44 (0.9%)
Back pain	14 (3%)	60 (3.7%)	157 (3.1%)
Pain in extremities	15 (3.2%)	57 (3.5%)	150 (3.0%)
Joint swelling	5 (1.1%)	19 (1.2%)	42 (0.8%)
Arthritis	8 (1.7%)	13 (0.8%)	57 (1.1%)
Subsultus	0 (0%)	14 (0.9%)	33 (0.7%)
Mobility disorder	7 (1.5%)	15 (0.9%)	61 (1.2%)
**General symptoms**	**25 (5.4%)**	**108 (6.6%)**	**357 (7.1%)**
Flu-like symptoms	5 (1.1%)	36 (2.2%)	105 (2.1%)
Dyspnoea	4 (0.9%)	10 (0.6%)	55 (1.1%)
Fever	2 (0.4%)	19 (1.2%)	43 (0.9%)
Nausea/vomiting	2 (0.4%)	18 (1.1%)	71 (1.4%)
Abdominal pain	1 (0.2%)	29 (1.8%)	84 (1.7%)
Fatigue	13 (2.8%)	51 (3.1%)	174 (3.5%)
Weakness	14 (3.0%)	52 (3.2%)	156 (3.1%)
Malaise	11 (2.4%)	45 (2.7%)	122 (2.4%)
**Neurological disorders**	**26 (5.6%)**	**102 (6.2%)**	**268 (5.4%)**
Headache	12 (2.6%)	60 (3.7%)	160 (3.2%)
Dizziness	11 (2.4%)	39 (2.4%)	121 (2.4%)
Paraesthesia	8 (1.7%)	28 (1.7%)	63 (1.3%)
Unconsciousness	0 (0%)	1 (0.1%)	6 (0.1%)
Neuralgia	6 (1.3%)	30 (1.8%)	79 (1.6%)
Seizure	1 (0.2%)	1 (0.1%)	7 (0.1%)
Minor stroke	0 (0%)	0 (0%)	4 (0.1%)
Paralysis	0 (0%)	2 (0.1%)	4 (0.1%)
Multiple sclerosis	0 (0%)	2 (0.1%)	5 (0.1%)
**Cardiovascular disorders/Risk factors**	**11 (2.4%)**	**41 (2.5%)**	**128 (2.6%)**
Diabetes	5 (1.1%)	4 (0.2%)	19 (0.4%)
Palpitations	3 (0.6%)	20 (1.2%)	63 (1.3%)
Chest pain	2 (0.4%)	16 (1.0%)	53 (1.1%)
Heart attack	0 (0%)	1 (0.1%)	2 (0%)
Myocarditis	0 (0%)	1 (0.1%)	4 (0.1%)
Vasculitis	1 (0.2%)	3 (0.2%)	3 (0.1%)
Pulmonary embolism	0 (0%)	0 (0%)	1 (0%)
Blood clot	1 (0.2%)	3 (0.2%)	4 (0.1%)
Coagulation disorder	2 (0.4%)	3 (0.2%)	6 (0.1%)
** Unsolicited health problems**	**38 (8.2%)**	**162 (9.9%)**	**389 (7.8%)**

Bold values indicate the number of persons with at least one of the following symptoms in the respective symptom-subgroup

Frequency of medical consultation, health problems and their perception 40 to 56 days after boost COVID-19 vaccination (long-term survey) with ChAdOx1 or mRNA vaccine in cohorts of participants with homologous (mRNA/mRNA or ChAdOx1/ChAdOx1) or heterologous (ChAdOx1/mRNA) prime-boost COVID-19 vaccination regimen who completed the long-term survey. Epilepsy and apoplexy were solicited but not reported by any participant. ChAdOx1: ChAdOx1 (AstraZeneca). mRNA: BNT162b2 (BioNTech/Pfizer) or mRNA-1273 (Moderna)

More participants in the mRNA/mRNA group reported long-term consequences (ChAdOx1/ChAdOx1 30% vs. ChAdOx1/mRNA 30% vs. mRNA/mRNA 38%). In the ChAdOx1/ChAdOx1 cohort participants reported most frequently that the vaccination was not comparable to previous vaccinations. Most health problems were unsolicited conditions, followed by musculoskeletal disorders in ChAdOx1/ChAdOx1 and ChAdOx1/mRNA cohort and by general conditions in mRNA/mRNA cohort, respectively. Results of the long-term survey are depicted in Table 3.

### Analysis by age and gender

#### Short-term survey

**Fig. 3:**
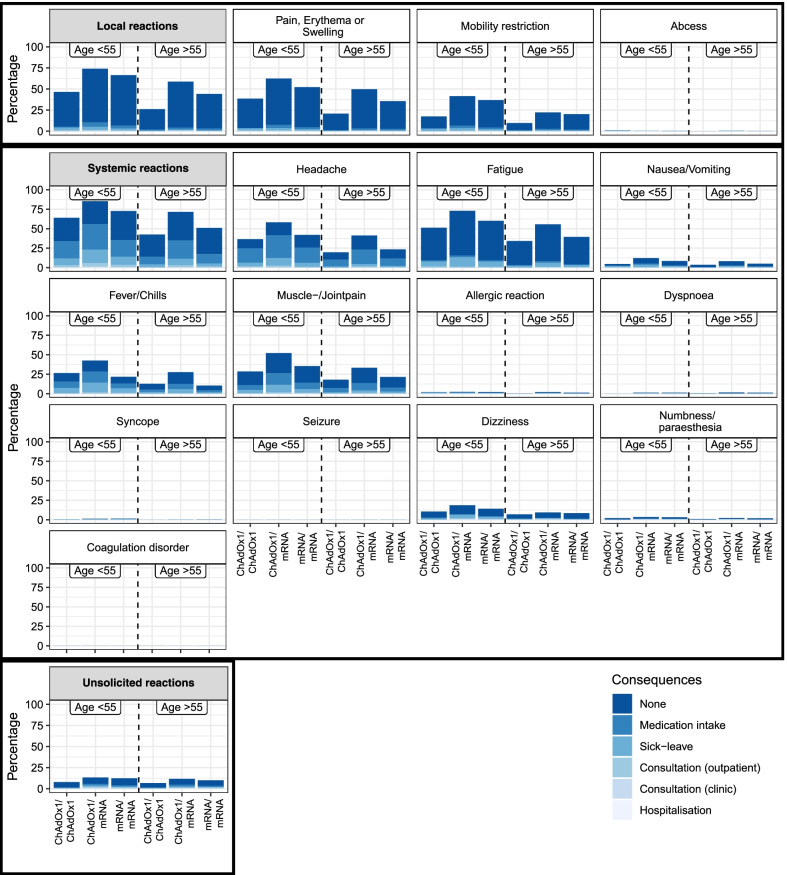
Reactions after boost COVID-19 vaccination in a homologous or heterologous regimen separated by age group. Solicited and unsolicited reactions 14 to 19 days after boost COVID-19 vaccination in participants with homologous (mRNA/mRNA or ChAdOx1/ChAdOx1) or heterologous (ChAdOx1/mRNA) prime-boost vaccination regimen with consequences in hierarchical order, as multiple choice was possible. The consequence perceived as most serious is reported (from no consequence to medication intake, sick leave, outpatient (practice) consultation, clinic (ambulant) consultation, and hospitalisation). Local reactions are a composite of pain, erythema, or swelling, mobility restriction, and abscess. Systemic reactions are a composite of the reactions from headache to coagulation disorder. ChAdOx1: ChAdOx1 (AstraZeneca). mRNA: BNT162b2 (BioNTech/Pfizer) or mRNA-1273 (Moderna)

**Fig. 4:**
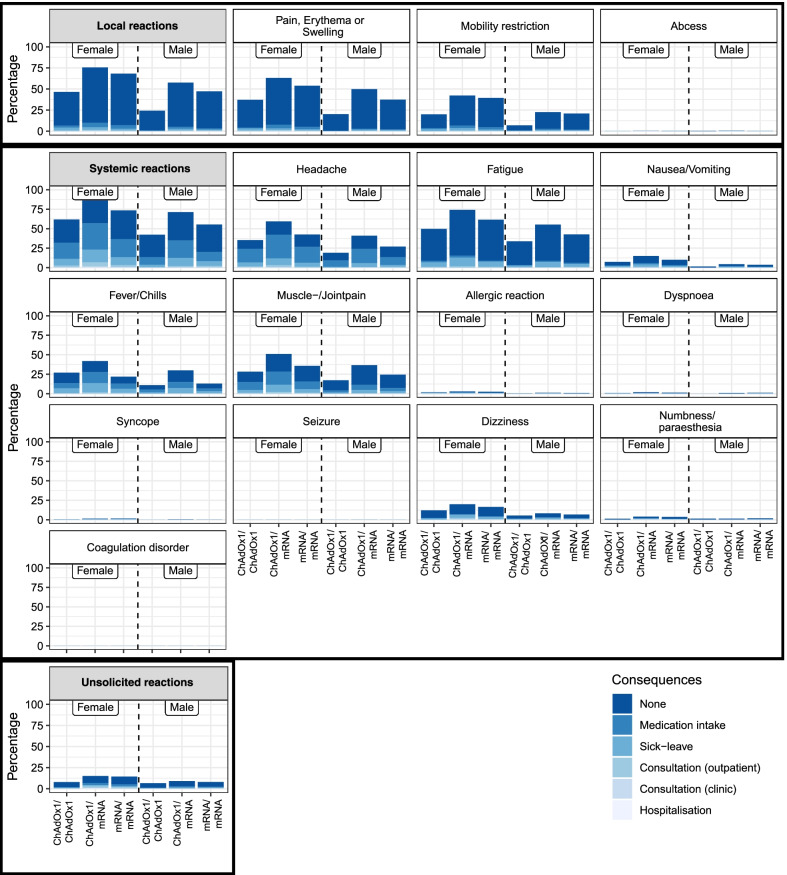
Reactions after boost COVID-19 vaccination in a homologous or heterologous regimen separated by gender. Solicited and unsolicited reactions 14 to 19 days after boost COVID-19 vaccination in participants with homologous (mRNA/mRNA or ChAdOx1/ChAdOx1) or heterologous (ChAdOx1/mRNA) prime-boost vaccination regimen with consequences in hierarchical order, as multiple choice was possible. The consequence perceived as most serious is reported (from no consequence to medication intake, sick leave, outpatient (practice) consultation, clinic (ambulant) consultation, and hospitalisation). Local reactions are a composite of pain, erythema, or swelling, mobility restriction, and abscess. Systemic reactions are a composite of the reactions from headache to coagulation disorder. ChAdOx1: ChAdOx1 (AstraZeneca). mRNA: BNT162b2 (BioNTech/Pfizer) or mRNA-1273 (Moderna)

Participants younger than 55 years did show an overall higher reactogenicity in the comparison of age groups (Fig. 3). The logistic regression confirmed an association of younger age with higher reactogenicity (OR = 0.964, 95% CI [0.960, 0.968]). Female participants showed an overall higher reactogenicity than male participants in the descriptive results and the logistic regression (OR = 2.23, 95% CI [1.997, 2.491], see Fig. 4).

#### Long-term survey

Older age was associated with reporting more medical consultations in the ChAdOx1/ChAdOx1 and ChAdOx1/mRNA cohort, whereas in the mRNA/mRNA cohort no differences were observed between the two age groups (< 55 years vs. ≥ 55 years: ChAdOx1/ChAdOx1 13 vs. 16%, ChAdOx1/mRNA 17 vs. 20%, mRNA/mRNA 16 vs. 16%, OR = 1.010, 95% CI [1.005, 1.014], see Table 4). Female patients reported a medical consultation more frequently in all cohorts (Female vs. male: ChAdOx1/ChAdOx1 16 vs. 14%, ChAdOx1/mRNA 20 vs. 12%, mRNA/mRNA 19% vs. 12%, OR = 1.859, 95% CI [1.620, 2.137], see Table 5).

**Table 4:** Medical consultation 40 to 56 days after boost COVID-19 vaccination separated by age group

	Age < 55 years			Age ≥ 55 years		
	ChAdOx1/ ChAdOx1	ChAdOx1/ mRNA	mRNA/ mRNA	ChAdOx1/ ChAdOx1	ChAdOx1/ mRNA	mRNA/ mRNA
	(*n* = 167)	(*n* = 1199)	(*n* = 3204)	(*n* = 295)	(*n* = 439)	(*n* = 1800)
**Medical consultation, thereof...**	**21 (12.6%)**	**198 (16.5%)**	**507 (15.8%)**	**48 (16.3%)**	**89 (20.3%)**	**289 (16.1%)**
**Outpatient**						
Yes	19 (90.5%)	174 (87.9%)	425 (83.8%)	42 (87.5%)	74 (83.1%)	242 (83.7%)
Planned	2 (9.5%)	24 (12.1%)	76 (15.0%)	4 (8.3%)	12 (13.5%)	41 (14.2%)
No	0 (0%)	0 (0%)	6 (1.2%)	2 (4.2%)	3 (3.4%)	6 (2.1%)
**Inpatient/Clinic**						
Yes	3 (14.3%)	13 (6.6%)	49 (9.7%)	10 (20.8%)	8 (9.0%)	36 (12.5%)
Planned	0 (0%)	1 (0.5%)	3 (0.6%)	1 (2.1%)	1 (1.1%)	6 (2.1%)
No	18 (85.7%)	184 (92.9%)	455 (89.7%)	37 (77.1%)	80 (89.9%)	247 (85.5%)
**Pre-existing conditions**						
Yes	2 (9.5%)	30 (15.2%)	61 (12%)	9 (18.8%)	10 (11.2%)	42 (14.5%)
Partially	6 (28.6%)	67 (33.8%)	171 (33.7%)	16 (33.3%)	28 (31.5%)	120 (41.5%)
No	13 (61.9%)	101 (51.0%)	275 (54.2%)	23 (47.9%)	51 (57.3%)	127 (43.9%)
**Suspected association to vaccination**						
Yes	1 (4.8%)	13 (6.6%)	27 (5.3%)	2 (4.2%)	3 (3.4%)	9 (3.1%)
Partially	5 (23.8%)	43 (21.7%)	116 (22.9%)	5 (10.4%)	17 (19.1%)	55 (19%)
No	15 (71.4%)	142 (71.7%)	364 (71.8%)	41 (85.4%)	69 (77.5%)	225 (77.9%)

Health problems reported in the long-term survey 40–56 days after boost COVID-19 vaccination with ChAdOx1 or mRNA vaccine (BNT162b2 or mRNA-1273) in cohorts of participants with homologous (mRNA/mRNA or ChAdOx1/ChAdOx1) or heterologous (ChAdOx1/mRNA) prime-boost COVID-19 vaccination regimen who completed the long-term survey

**Table 5:** Medical consultation 40 to 56 days after boost COVID-19 vaccination separated by gender

		Female			Male	
	ChAdOx1/ ChAdOx1	ChAdOx1/ mRNA	mRNA/ mRNA	ChAdOx1/ ChAdOx1	ChAdOx1/ mRNA	mRNA/ mRNA
	(*n* = 205)	(*n* = 1189)	(*n* = 2865)	(*n* = 257)	(*n* = 448)	(*n* = 2133)
**Medical consultation, thereof...**	**33 (16.1%)**	**233 (19.6%)**	**533 (18.6%)**	**36 (14%)**	**53 (11.8%)**	**261 (12.2%)**
**Outpatient**						
Yes	32 (97.0%)	205 (88.0%)	453 (85.0%)	29 (80.6%)	42 (79.2%)	213 (81.6%)
Planned	1 (3.0%)	27 (11.6%)	75 (14.1%)	5 (13.9%)	9 (17.0%)	41 (15.7%)
No	0 (0%)	1 (0.4%)	5 (0.9%)	2 (5.6%)	2 (3.8%)	7 (2.7%)
**Inpatient/Clinic**						
Yes	5 (15.2%)	16 (6.9%)	44 (8.3%)	8 (22.2%)	5 (9.4%)	41 (15.7%)
Planned	0 (0%)	2 (0.9%)	5 (0.9%)	1 (2.8%)	0 (0%)	4 (1.5%)
No	28 (84.8%)	215 (92.3%)	484 (90.8%)	27 (75.0%)	48 (90.6%)	216 (82.8%)
**Pre-existing conditions**						
Yes	2 (6.1%)	28 (12.0%)	60 (11.3%)	9 (25.0%)	12 (22.6%)	43 (16.5%)
Partially	10 (30.3%)	85 (36.5%)	217 (40.7%)	12 (33.3%)	10 (18.9%)	73 (28.0%)
No	21 (63.6%)	120 (51.5%)	256 (48%)	15 (41.7%)	31 (58.5%)	145 (55.6%)
**Suspected association to vaccination**						
Yes	1 (3%)	16 (6.9%)	26 (4.9%)	2 (5.6%)	0 (0%)	10 (3.8%)
Partially	4 (12.1%)	49 (21%)	118 (22.1%)	6 (16.7%)	11 (20.8%)	53 (20.3%)
No	28 (84.8%)	168 (72.1%)	389 (73%)	28 (77.8%)	42 (79.2%)	198 (75.9%)

Health problems reported in the long-term survey 40 to 56 days after boost COVID-19 vaccination with ChAdOx1 or mRNA vaccine in cohorts of participants with homologous (mRNA/mRNA or ChAdOx1/ChAdOx1) or heterologous (ChAdOx1/mRNA) prime-boost COVID-19 vaccination regimen who completed the long-term survey. ChAdOx1: ChAdOx1 (AstraZeneca). mRNA: BNT162b2 (BioNTech/Pfizer) or mRNA-1273 (Moderna)

## Discussion

A higher reactogenicity was reported with the heterologous vaccination regimen as compared to homologous regimens 14 to 19 days after the second COVID-19 vaccination. A medical consultation was reported by a comparable proportion with all three regimens 40 to 56 days after the second COVID-19 vaccination. Female gender was associated with a higher rate of reported reactions and medical consultations, respectively. In the short-term follow-up, younger age was associated with a higher rate of reported reactions, whereas in the long-term follow-up elderly people in ChAdOx1/ChAdOx1 and ChAdOx1/mRNA reported more medical consultations.

Previous studies showed that the heterologous regimen is tolerable and no serious adverse event occurred that were suspected to be due to the vaccination [21, 31]. However, the heterologous boost with mRNA leading to a higher reactogenicity is consistent with results of two studies from the UK [16, 18]. In these studies, the interval between prime and boost did not differ (widely) between the regimens. In contrast, two studies from Germany reported a largely comparable reactogenicity after boost with mRNA vaccine in heterologous and homologous regimens [19, 20]. Hillus et al. reported even a slight increase in systemic reactions after homologous mRNA (BNT162b2) boost compared to heterologous ChAdOx1/mRNA or homologous ChAdOx1/ChAdOx1 boost. In both studies, the mean interval between prime and boost vaccination differed largely with a longer interval in heterologous regimen [19]. The authors hypothesized that the extended interval in the heterologous cohort as compared to the homologous regimens with shorter intervals could have led to a decrease in reactogenicity in the heterologous group [19]. In our study, the difference between the intervals in the different cohorts was smaller, because since April 2021, a six-week interval was recommended in Germany for the homologous mRNA regimen instead of the initial three weeks interval. This was due to limited vaccine resources and a higher immunogenicity with a longer interval [8, 32]. It might be assumed, that the reactogenicity is higher with a heterologous mRNA boost but higher reactogenicity might also be associated with a smaller interval between the first and second dose of COVID-19 vaccination.

Female gender was associated with higher reactogenicity not only in our study but also in Powell et al. and Borobia et al. [16, 17]. This association was previously shown for a lot of virus vaccines other than COVID-19 along with a higher immunogenicity in female individuals [33]. Female participants also reported medical consultations in the long-term follow-up more frequently. This association as well does not seem to be specific for COVID-19 vaccinations. A scoping review revealed that female gender was frequently associated with an increased medical health care utilization in general [34]. Not only female gender but also younger age seems to be associated with a higher reactogenicity. Age as an influencing factor on the reporting of adverse events was also mentioned in other studies on COVID-19 vaccination. Similar to our results, studies showed that younger age was associated with the reporting of a higher reactogenicity [1, 2, 16, 17].

However, medical consultations in the long-term survey were reported more frequently in participants aged 55 years or older. As comorbidities were more common in this age group, the higher rate of medical consultations can possibly rather be attributed to participants’ comorbidities than to the vaccination. This hypothesis might be strengthened by the finding that the proportion of medical consultation reported in the long-term survey was comparable between the regimens. Additionally, participants assumed an association between the second vaccination and health problems in the long-term survey less frequently as compared to an association between the second vaccination and reactions in the short-term survey.

## Limitations

Participants were recruited on the day of vaccination to reduce selection bias. However, some participants registered with delay to their vaccination, possibly due to their reactions or health problems. As there was no higher rate of reported reactions in participants registered with delay, this potential bias is probably small. Since all information is given by the vaccinated persons themselves, certain groups of individuals (e.g. seriously ill, cognitively impaired) were not able to participate. This narrows generalizability of the results to some extent. Due to recommendations in vaccination strategy in Germany at the time of recruitment, characteristics of the groups differ in regard to age, gender, and vaccination interval, as well as in total size of study population. Therefore, subgroup analyses were performed separately for age and gender.

## Conclusion

The reactogenicity of the heterologous regimen seems to be higher compared to homologous regimens. Until now, however, there is no signal that severe adverse events are more common with this regimen, although further research is necessary. Other factors like a higher efficacy and limited resources during the pandemic might overweight a tolerable higher reactogenicity in the recommendations of specific regimens.

## Data Availability

Aggregated data that support the findings of this study are available from the corresponding author for researchers who provide a methodologically sound proposal after consent of the data protection supervisor.
